# Are Hair Scalp Trace Elements Correlated with Atherosclerosis Location in Coronary Artery Disease?

**DOI:** 10.1007/s12011-024-04335-w

**Published:** 2024-08-15

**Authors:** Tomasz Urbanowicz, Anetta Hanć, Julia Frąckowiak, Maksymilian Białasik-Misiorny, Anna Olasińska-Wiśniewska, Beata Krasińska, Aleksandra Krasińska-Płachta, Jolanta Tomczak, Mariusz Kowalewski, Zbigniew Krasiński, Andrzej Tykarski, Marek Jemielity

**Affiliations:** 1 Cardiac Surgery and Transplantology Department, Poznan University of Medical Sciences, Dluga ½ Street, 61-701 Poznan, Poland; 2 Thoracic Research Centre, Innovative Medical Forum, Collegium Medicum Nicolaus Copernicus University, Bydgoszcz, Poland; 3 Department of Trace Analysis, Faculty of Chemistry, Adam Mickiewicz University, 61-614 Poznan, Poland; 4 Poznan University of Medical Sciences, 61-701 Poznan, Poland; 5 Department of Hypertensiology, Angiology and Internal Medicine, Poznan University of Medical Sciences, 61-701 Poznan, Poland; 6 Department of Ophthalmology, Poznan University of Medical Sciences, 61-107 Poznan, Poland; 7 Department of Cardiac Surgery and Transplantology, Ministry of Interior and Administration, National Medical Instituteof the , Warsaw, Poland; 8 Cardio-Thoracic Surgery Department, Heart and Vascular Centre, Maastricht University Medical Centre (MUMC), Cardiovascular Research Centre Maastricht (CARIM), Maastricht, the Netherlands; 9 Department of Vascular, Endovascular Surgery, Angiology, and Phlebology Medical University, Poznan University of Medical Science, 61-701 Poznań, Poland

**Keywords:** Coronary disease, Atherosclerosis location, Trace elements, Hair, Scalp, ICP-MS

## Abstract

Coronary artery disease is among the leading current epidemiological challenges. The genetic, clinical, and lifestyle-related risk factors are well documented. The reason for specific epicardial artery locations remains unsolved. The coronary artery topography and blood flow characteristics may induce local inflammatory activation. The atherosclerotic plaque formation is believed to represent inflammatory response involving enzymatic processes co-factored by trace elements. The possible relation between trace elements and coronary artery disease location was the subject of the study. There were 175 patients (107 (61) men and 68 (39) females) in a median (Q1-3) age of 71 years (65–76) admitted for coronary angiography due to chronic coronary syndrome. The angiographic results focused on the percentage of lumen stenosis in certain arteries and were compared with the results for hair scalp trace elements. The correlation between left main coronary artery atherosclerotic plaques and nickel (Ni), zinc (Zn), and antimony (Sb) hair scalp concentration was noted. The analysis revealed a positive relation between left descending artery disease and chromium (Cr), sodium (Na), arsenic (As), and molybdenum (Mo) and a negative correlation with strontium (Sr). The atherosclerotic lesion in the circumflex artery revealed correlations in our analysis with sodium (Na), potassium (K), chromium (Cr), nickel (Ni), arsenic (As), and negative with strontium (Sr) (*r*) hair scalp concentrations. The negative correlations between right coronary artery disease and magnesium (Mg) and strontium (Sr) concentrations were noted. The possible explanation of different epicardial artery involvement and severity by atherosclerotic processes may lay in their topography and blood rheological characteristics that induce different inflammatory reactions co0factored by specific trace elements. The trace element concentration in the hair scalp may correlate with a particular coronary atherosclerotic involvement, including the severity of lumen reduction. This may indicate the missing link between the pathophysiological processes of atherosclerosis development and its location in coronary arteries.

## Introduction

Coronary artery disease is among one of the leading current epidemiological challenges. The genetic, clinical, and lifestyle-related risk factors are well documented. The current guidelines highlight effective therapies’ significance for comorbidities [1]. Preventive treatment is claimed to be effective in reducing events but not eliminating them. The atherosclerotic lesion formation is the effect of multiple reactions. It is believed to represent chronic inflammatory disease mediated by proinflammatory cytokines, active lipids, inflammatory signalling pathways, and adhesion molecules [2]. The non-critically stenotic coronary lesions are claimed to provoke major acute coronary events. The plaque characteristics based on local anatomy and biomechanical content are the subject of current investigations [3]. The previous studies indicated the upregulation of reactive oxygen species, which may exacerbate reactive oxygen species generation and induce inflammatory reactions about trace elements, resulting in endothelial dysfunction and lipid metabolism distribution [4]. The main “battlefield” in atherosclerotic disease is the intima of the artery, where structural changes and parafunctional reactions lead to abnormal deposition of lipids, blood components, and fibrous tissue at the site of injury, which slowly but inexorably changes the architecture of the arterial wall [5]. Elemental analysis plays a role in understanding chronic inflammatory disease affecting the arteries.

The origin of atherosclerotic lesions in different epicardial arteries remains unknown, but artery topography and secondary blood flow characteristics may initiate separate interactions between vascular endothelium and inflammatory cells, as suggested in previous studies [6]. Within the same artery, the specific locations due to the interaction between endothelial function and blood flow characteristics make them more prone to atherosclerotic plaque development and propagation, according to previous reports [7].

**Fig. 1:**
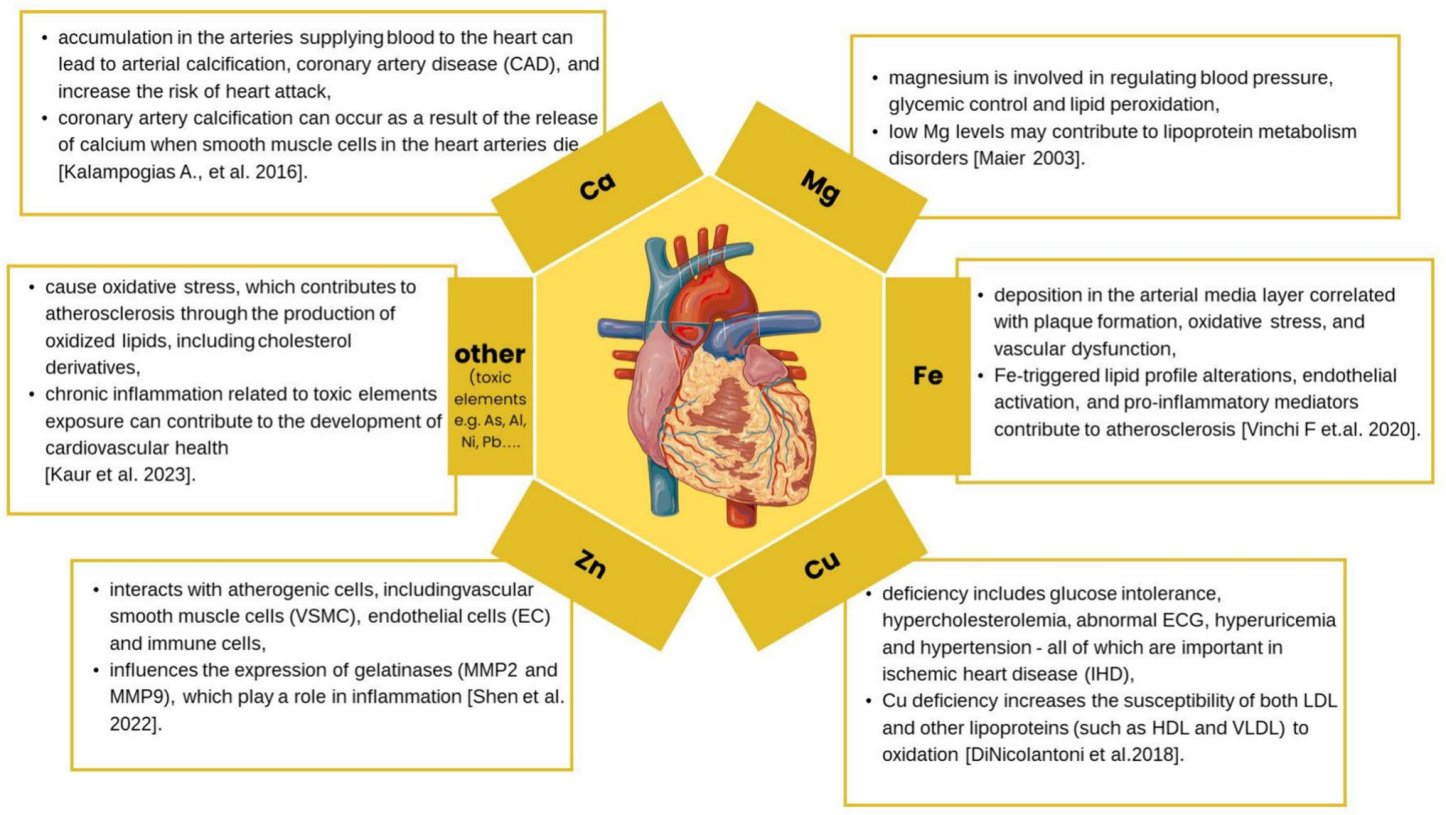
The importance of selected elements in the atherosclerotic process. Parts of the figure were drawn using pictures from Server Medical Art. Servier Medical Art by Servier is licensed under a Creative Commons Attribution 3.0 Unported License

Several trace elements, such as calcium (Ca), magnesium (Mg), copper (Cu), zinc (Zn), and others, perform significant functions in the human body, and any disturbance of their homeostasis affects the disease process. Figure 1 shows the importance of selected elements in the process of atherosclerosis. Previous bioimaging [8] and ultrastructural analyses of arteries [5] revealed the presence of mature and stable amorphous calcium phosphate deposits in tissues affected by atherosclerosis. Ca, lead (P), Mg, strontium (Sr), Pb, and barium (Ba) were determined in the atherosclerotic plaque and its immediate vicinity. A higher Zn content was defined in the area connecting the arterial wall with the atherosclerotic plaque, which results from the activity of matrix metalloproteinases in the arterial wall.

Testing elements in the blood is a commonly used method, which may be subject to uncertainty because the content of elements in the blood is influenced by both the on-going inflammation and many other factors, such as the time of day, serum protein concentration, age, gender, and the type of meals consumed. The level of elements/chemicals in the blood often reflects exposure over the last few hours or days. Therefore, an exciting alternative may be examining elements in hair collected from patients, which has been adequately prepared for elemental analysis. Determining the content of elements in hair allows us to avoid daily changes in the content because it does not depend on homeostasis. Compared to hair, the concentration of elements in the blood often does not correspond to their content in the body because the homeostasis mechanism regulates their concentration. Therefore, hair analysis may be an exciting way of conducting long-term assessments.

The chronic process of atherosclerotic plaque formation is asymptomatic. The upregulation of expression of scavenger receptors occurs secondary to proinflammatory stimuli and results in free and esterified cholesterol deposition in macrophages, eventually allowing for foam cell formation [9]. Inflammation is initiated by innate immune reactions to modified lipoproteins and is perpetuated by T helper lymphocyte 1 (TH1) that reacts to autoantigens from the apolipoprotein B100 protein of low-density lipoprotein (LDL) [10]. Metabolic transitions of macrophages described in the atherosclerotic progression contribute to lesion initiation and continue by advanced lesions characterised by necrotic cores and proceed into lesion regression following aggressive lipid-lowering [11]. Recent studies indicate that the main contributor to atherosclerotic plaque formation is affiliated arterial smooth muscle cells (SMCs), the source of most foam cells [12]. The possible explanation of the plaque appearance site is likely related to the interactions between SMCs and macrophages. In animal studies, the cell transcriptome analyses pointed out the role of vascular smooth muscle cells (VSMCs) that can differentiate into multiple lineages in plaques [13].

The risk factors modification and control are related to the coronary bed, which does not focus on the particular artery. There is no possible explanation indicating the formation of the atherosclerotic plaque. According to current knowledge, the aetiology of the actual location is unknown and unpredictable.

The study aimed to find possible markers of atherosclerosis location and severity of arterial lumen narrowing in epicardial beds related to trace element hair scalp concentration. Macro and microelements are involved in many cellular and physiological processes, performing catalytic, regulatory and signalling functions. Disturbances in the balance of these elements may cause dysfunctions in the cardiovascular system.

## Patients and Methods

There were 175 patients (107 (61) men and 68 (39) females) in a median (Q1-3) age of 71 years (65–76) admitted for coronary angiography due to chronic coronary syndrome. They presented with mean Canadian Cardiovascular Society (CCS) class 2.1 ± 0.7, including 89 patients with angina equivalent. Patients were characterized by the co-existence of arterial hypertension (*n* = 155 (89)), dyslipidaemia (*n* = 160 (91)), diabetes mellitus (*n* = 67 (38)), stroke (*n* = 13 (7)), peripheral artery disease (PAD) (*n* = 40 (23)) and chronic obstructive pulmonary disease (COPD) (*n* = 13 (7)). There were 36 (21) active smokers, and 89 more were presenting the history of nicotinism. The patients’ median (Q1–Q3) values of height and weight were 168 cm (161–176) and 84 kg (70–95), respectively.

### Methods

All patients were admitted to the internal medicine department after the cardiologist’s referral. The hair scalp samples were taken on admission before cine angiography. After routine evaluation, coronary angiography was performed for epicardial atherosclerosis estimation. Not only the type of coronary artery but also the lumen narrowing by atherosclerotic plaques were examined and taken into further evaluation.

### Hair Scalp Methodology

Hair without perm or dye, 2–3 cm long, was cut from the occipital part of the head, close to the skin. The collected hair samples were washed and dried according to the procedure described [14]. A dry sample of 150–200 mg of hair was mineralised in the DigiTube system (SCP Science Ltd., Canada). Four millilitres of 65% nitric acid (Suprapure, Merc, Germany) and 1 ml of 30% hydrogen peroxide (Supelco, Merc, Germany) were used for mineralisation. Such prepared samples were heated at 150 °C for 4 h. After cooling the samples to room temperature, the samples were diluted 100-fold with Mili-Q water (Millipore Direct Q-3, Merc, Germany). The samples prepared in this way were analysed for the content of elements using the SN-ICP-MS method (7700x Agilent, USA) by the previously described procedure by Urbanowicz et al. [15]. The validity of the analytical method was assessed by analysing the certified reference material (CRM), NCS ZC 81002b Human Hair (Beijing, China). Trueness was evaluated by applying the certified reference material and expressed as recovery values (%) ranging from 94 to 107%.

### Statistical Analysis

The normality of the distribution of variables was tested with the Shapiro–Wilk test. The *t* test, Cochran-Cox test, Mann–Whitney test and Fisher’s exact test were used where applicable to compare the variables between two groups. Spearman’s correlation analysis was used to describe the correlation between the variables. Statistical analysis was performed using Statistica 13 by TIBCO. *p* < 0.05 was considered statistically significant.

### Bioethics Committee Approval

The study was performed according to the principles of Good Clinical Practice and the Declaration of Helsinki. It was approved by the Local Ethics Committee of the Medical University of Poznan (approval number: 875/22 on 3 November 2022). All patients gave their informed consent for inclusion in the study.

## Results

### Coronary Angiography Results

Coronary angiographies were performed by the same experienced team in the reference centre using left radial access. There were 39 (22%) patients presenting left central coronary artery atherosclerosis, including significant disease (> 50% lumen narrowing) in 19 (11%) patients. In 146 (83%) patients, no plaques were detected in the left main. The atherosclerotic plaques in LAD were detected in 101 (58%) patients. Significant (lumen narrowing > 70%) stenosis was found in 60 (34%) patients. In Cx angiography, the atherosclerotic plaques, including significantly stenotic, were found in 72 (41%) and 41 (23%), respectively. The atherosclerotic plaques were found in RCA angiograms in 81 (44%) patients, and significant stenosis was found in 44 (25%). The detailed information is presented in Table 1.

**Table 1:** Atherosclerosis results in performed coronary angiographies

	LMCA *n* = 39	LAD *n* = 101	Cx *n* = 72	RCA *n* = 81
Normal results (*n* (%))	146 (79)	84 (45)	113 (61)	104 (56)
Atherosclerotic plaques (*n* (%))	39 (21)	101 (55)	72 (39)	81 (44)
Significant stenosis (*n* (%))	19 (10)	60 (32)	41 (22)	41 (22)
Artery occlusion (*n* (%))	0 (0)	11 (6)	2 (1)	9 (5)

*Cx* circumflex artery, *LAD* left descending artery, *LMCA* left main coronary artery, *RCA* right coronary artery disease

Based on angiograms, they were divided into two groups composed of patients presenting any coronary artery disease on angiograms (*n* = 116) and normal epicardial arteries (*n* = 59). There were no statistical differences regarding demographical or clinical characteristics, including pharmacotherapy or admission, as presented in Table 2.

**Table 2:** Patient characteristics representing any coronary disease and normal angiograms

	Any disease in epicardial arteries (2) *n* = 116	Normal angiogram in any epicardial artery (1) *n* = 59	*p*
Demographical:			
Age (years) (median, Q1–Q3)	72 (65—76)	71 (65 – 75)	0.434
Sex (male/female) (*n*)	70 / 46	37 / 22	0.604
Weight (kg) (median, Q1–Q3)	83 (71 – 95)	95 (70 – 94)	0.801
Height (cm) (median, Q1–Q3)	168 (160 – 176)	164 (161 – 175)	0.745
Co-morbidities:			
Arterial hypertension (*n*, %)	101 (87)	54(92)	0.458
Dyslipidaemia (*n*, %)	105 (91)	55 (93)	0.776
Diabetes mellitus (*n*, %)	46 (40)	21 (36)	0.626
Stroke (*n*, %)	8 (7)	5 (9)	0.766
Peripheral artery disease (*n*, %)	26 (22)	14 (24)	0.851
COPD (*n*, %)	9 (8)	4 (7)	1.000
Nicotinism:			
Active (*n*, %)	24 (21)	12 (21)	1.000
In history (*n*, %)	62 (53)	27 (46)	0.625
Pharmacotherapy:			
B-blockers (*n*, %)	114 (98)	59 (100)	0.551
ASA (*n*, %)	116 (100)	59 (100)	1.000
Statins (*n*, %)	105 (91)	55 (93)	0.776
ACE-I (*n*, %)	93 (80)	45 (76)	0.562
CCB (*n*, %)	38 (33)	16 (27)	0.492
Diuretics (*n*, %)	12 (10)	7 (12)	0.800

*ASA* aspirin, *B-blockers* beta-adrenergic receptors blockers, *CCB* calcium channel blockers, *cm* centimetres, *COPD* chronic obstructive pulmonary disease, *kg* kilograms, *Q* quartiles

There were 139 (79%) percutaneous coronary interventions (PCIs) performed due to significant coronary artery disease, including 16 (9%) PCI-LMCA, 20 (11%) PCI-LAD, 15 (9%) PCI-Cx and 6 (3%) PCI-RCA. The epicardial artery occlusion was noted in 22 (13%) patients. The median width and length of implanted stents were 2.3 (1.5–3.0) mm and 18 (14–24) mm, respectively.

### Hair scalp Trace Elements Results

The mineral hair scalp concentrations were evaluated and are presented in Table 3, indicating significant differences in magnesium (Mg) (*p* < 0.001), potassium (K) (*p* = 0.006) and calcium (K) (*p* < 0.001), vanadium (V) (*p* = 0.004), chrome (cr) (*p* = 0.006), arsenic (As) (*p* = 0.006) and strontium (Sr) (*p* < 0.001).

**Table 3:** Hair scalp element concentrations (mg/kg)

Trace elements	Concentration Whole group *n* = 175	Any disease in epicardial arteries (2) *n* = 116	Normal angiogram in any epicardial artery (1) *n* = 59	*p*
Li Na Mg Al K Ca Ti V Cr Mn Fe Co Ni Cu Zn As Se Rb Sr Mo Cd In Sb Cs Ba Pb U	0.018 (0.005–0.042) 249 (166–390) 21.8 (12.6–40.6) 5.3 (3.3–9.1) 8.5 (5.1–16.9) 170 (70–689) 0.345 (0.234–0.508) 0.020 (0.000–0.031) 0.965 (0.638–1.507) 0.208 (0.135–0.318) 10.9 (8.8–15.3) 0.022 (0.014–0.036) 0.817 (0.599–1.308) 13.3 (10.8–19.6) 155 (124–171) 0.033 (0.023–0.052) 0.440 (0.352–0.521) 0.016 (0.005–0.036) 0.536 (0.206–1.556) 0.120 (0.090–0.182) 0.013 (0.006–0.027) 0.008 (0.003–0.020) 0.025 (0.015–0.046) 0.011 (0.004–0.025) 0.713 (0.301–1.547) 0.212 (0.109–0.491) 0.007 (0.004–0.015)	0.018 (0.005–0.042) 272 (188–422) 18.1 (12.0–31.0) 5.3 (3.3–9.0) 9.4 (6.-3–20.1) 126 (62–379) 0.353 (0.235–0.512) 0.026 (0.000–0.032) 1.010 (0.715–1.723) 0.193 (0.140–0.299) 11.7 (9.2–15.9) 0.022 (0.014–0.031) 0.888 (0.612–1.432) 12.7 (10.9–17.4) 155 (119–171) 0.035 (0.023–0.055) 0.446 (0.359–0.514) 0.018 (0.006–0.038) 0.408 (0.172–0.965) 0.131 (0.095–0.199) 0.012 (0.005–0.026) 0.010 (0.004–0.021) 0.024 (0.016–0.047) 0.012 (0.005–0.025) 0.520 (0.284–1.270) 0.185 (0.100–0.444) 0.007 (0.003–0.015)	0.05 (0.05–0.036) 208 (149–315) 33.0 (14.5–97.2) 5.1 (3.5–10.2) 6.9 (4.0–11.9) 333 (121–1612) 0.315 (0.230–0.502) 0.015 (0.000–0.025) 0.784 (0.561–1.220) 0.237 (0.132–0.383) 10.1 (8.7–13.1) 0.026 (0.012–0.050) 0.724 (0.559–1.119) 14.4 (11.1–25.5) 159 (125–171) 0.028 (0.013–0.040) 0.412 (0.341–0.539) 0.011 (0.004–0.031) 1.171 (0.329–4.638) 0.098 (0.081–0.146) 0.015 (0.007—0.034) 0.007 (0.003–0.017) 0.026 (0.013 0.045) 0.008 (0.003–0.023) 0.997 (0.510–2.069) 0.301 (0.152–0.576) 0.010 (0.004–0.015)	0.982 0.009 < 0.001 0.723 0.006 < 0.001 0.484 0.004 0.006 0.259 0.073 0.590 0.054 0.071 0.948 0.006 0.878 0.116 < 0.001 0.002 0.230 0.211 0.911 0.093 0.004 0.041 0.612

*Al* aluminium, *As* arsenic *Ba* barium, *Ca* calcium, *Cd* cadmium, *Co* cobalt *Cr* chromium, *Cs* caesium, *Cu* copper *Fe* ferrum, *In* indium, *K* potassium *Li* lithium, *Mg* magnesium, *Mn* manganese, *Mo* molybdenum, *Na* sodium, *Ni* nickel, *Pb* lead, *Rb* rubidium, *Sb* antimony, *Se* selenium, *Sr* strontium *Ti* titanium, *U* uranium, *V* vanadium, *Zn* zinc

### Correlations

The correlation between the severity of left main coronary artery lumen narrowing by atherosclerotic plaques and nickel (Ni) (*r* = 0.161, *p* = 0.033) followed by zinc (Zn) (*r* = 0.155, *p* = 0.041) and antimony (Sb) ( *r* = 0.162, *p* = 0.032) hair scalp concentration was noted.

The analysis revealed positive relation between severity of left-descending artery disease and hair scalp concentration of chromium (Cr) (*r* = 0.166, *p* = 0.028), sodium (Na) (*r* = 0.219, *p* = 0.004), arsenic (As) (*r* = 0.252, *p* < 0.001) and molybdenum (Mo) (*r* = 0.248, *p* < 0.001) and negative correlation with strontium (Sr) (*r* =  − 0.169, *p* = 0.025).

The severity of circumflex artery lumen stenosis revealed correlations in our analysis with sodium (Na) (*r* = 0.302, *p* < 0.001), potassium (K) (*r* = 0.247, *p* < 0.001), chromium (Cr) (*r* = 0.238, *p* = 0.002), nickel (Ni) (*r* = 0.200, *p* = 0.008) and arsenic (As) (*r* = 0.180, *p* = 0.017) and negative with strontium (Sr) (*r*) hair scalp concentrations.

There were negative correlations between the percentage of lumen reduction in right coronary artery disease and magnesium (Mg) (*r* =  − 0.165, *p* = 0.029) and strontium (Sr) (*r* =  − 0.148, *p* = 0.050) concentrations.

The significant correlation between other laboratory parameters and coronary artery disease risk in detailed/particular epicardial arteries was performed as presented in Table 4.

**Table 4:** The correlation between hair scalp element concentrations and angiographic results of particular epicardial arteries

	LMCA *n* = 175	LAD *n* = 175	Cx *n* = 175	RCA/PDA *n* = 175
Na	0.079 0.319	0.219 0.004*	0.302 < 0.001*	− 0.013 0.866
Mg	0.024 0.750	− 0.149 0.051	− 0.129 0.089	− 0.165 0.029*
K	0.060 0.432	0.176 0.020	0.247 < 0.001*	0.015 0.847
Ca	− 0.031 0.688	− 0.136 0.074	− 0.229 0.002*	− 0.110 0.150
Cr	0.112 0.138	0.166 0.028*	0.238 0.002*	0.031 0.681
Fe	0.081 0.286	0.126 0.096	0.165 0.030*	− 0.043 0.573
Ni	0.161 0.033*	0.144 0.057	0.200 0.008*	− 0.016 0.834
Zn	0.155 0.041*	0.017 0.819	0.087 0.254	− 0.124 0.077
As	0.081 0.287	0.252 < 0.001*	0.180 0.017*	0.131 0.083
Rb	0.076 0.318	0.160 0.035*	0.147 0.052	− 0.006 0.933
Sr	0.006 0.939	− 0.169 0.025*	− 0.170 0.024*	− 0.148 0.050*
Mo	0.119 0.116	0.248 < 0.001*	0.271 < 0.001	0.043 0.574
Sb	0.162 0.032*	0.081 0.288	0.110 0.147	0.050 0.515

*Al* aluminium, *As* arsenic *Ba* barium, *Ca* calcium, *Cd* cadmium, *Co* cobalt, *Cr* chromium, *Cs* caesium, *Cu* copper, *Cx* circumflex artery, *Fe* ferrum, *In* indium, *K* potassium, *LAD* left descending artery, *Li* lithium, *LCA* left main coronary artery, *Mg* magnesium, *Mn* manganese, *Mo* molybdenum, *Na* sodium, *Ni* nickel, *Pb* lead, *RCA* right coronary artery, *Rb* rubidium, *PDA* posterior descending artery, *Sb* antimony, *Se* selenium, *Sr* strontium, *Ti* titanium, *U* uranium, *V* vanadium, *Zn* zinc

## Discussion

Our analysis points to the relation between hair scalp concentration trace elements and atherosclerotic plaque location, resulting in the severity of lumen stenosis in particular types of coronary arteries. The importance of the results of our study is based on, first, to the best of our knowledge, atherosclerotic plaques’ different locations related to the presented factors. As the trace elements are co-factors of many enzymatic processes, including redox reactions [16], the correlation of other components in various lesion locations may indicate their possible involvement in plaque formation. The possible explanation for topographic location within the left-sided coronary bed [17] may be explained, as presented in our study, by diverse inflammatory reactions factored by different trace elements. Previous reports indicated the relation between non-modifiable cardiovascular risk factors and left descending artery involvement [18]. Our analysis indicates the diverse trace elements increased concentration related to coronary artery topography but also signifies a relation between some trace elements and multiple arteries (nickel, chromium, sodium, strontium and arsenic) pointing out the possible shared pathways in lesion formation and progression. The results of our analysis may point out the differences in pathological processes that lead to location-related atherosclerosis development. Depending on various locations, different pathways are involved in atherosclerosis progression.

Our analysis suggested the relationship between zinc hair scalp concentration and severity of left main disease. The left main coronary represents the very beginning of the sided epicardial bed; the thorough evaluation of the possible risk factors is of utmost importance as haemodynamically significant lesions can be life-threatening conditions. The inflammatory processes that take place in this location that are accompanied by traditional risk factors can be related to inflammatory activation factored by certain trace elements such as zinc. Zinc belongs to trace elements involved in activating more than 300 enzymes, including its involvement in atherosclerosis by zinc-dependent monocyte ingestion of oxidized LDL to form foam cells [19]. There are contradictions in the results of the serum zinc analysis into coronary artery disease prediction [20, 21]. Our report could partially explain the dichotomous effects, that not the lesion but location is the key to understanding the atherosclerotic lesion pathophysiology. The misleading results may be explained by the location-related involvement of zinc in atherosclerosis pathophysiology. One of the zinc-dependent endopeptidases is matrix metalloproteinases (MMPs), which are secreted by many cells, including fibroblasts, vascular smooth muscle (VSM), and leukocytes [22]. They are involved in vascular tissue remodelling during angiogenesis. In their review, Shen et al. [19] pointed out the regulatory role of zinc on multiple cellular processes and its pivotal role in atherogenesis formation. Previous analyses also indicated significant associations between zinc concentrations and several coronary risk factors, and 10 years of coronary risk scores were calculated [23].

The damaging effect of chromium on endothelial function in vitro studies may justify the results of our study [24]. Our analysis implied the association between chromium hair scalp concentration and the percentage of lumen reduction in left-descending artery disease. The hair scalp chromium concentration in coronary disease was presented by Ilyas et al. [25]. The strong relationship between chromium and superoxide dismutase (SOD) implied cell injury in diabetic patients [26]. Hill et al. [27] revealed the pathological processes triggered by chromium exposure resulting in DNA-dependent protein kinase (DNA-PK)–mediated apoptosis. Our results suggest an association between chromium concentration and coronary artery disease in the left-descending artery and circumflex artery. However, the systemic review by Nigra et al. [28] did not support its role in cardiovascular diseases. In the study by Olcay et al. [24], an increased chromium concentration was detected in carotid endarterectomies compared to standard carotid samples.

Our analysis revealed the negative impact of magnesium concentration on right coronary artery plaque development measured by lumen reduction. The magnesium deficiency–dependent mechanisms of inflammatory activation are postulated phagocytic cell induction and nuclear factor (NF)-κB activation [29]. Its scarcity persuades lipoprotein metabolism. Magnesium deficiency is incipient as an endothelial dysfunction inducer [30]. Our analysis points out the adverse association between atherosclerosis development in the right coronary bed and low magnesium. Magnesium potentiates the production of local vasodilatory mediators (prostacyclin and nitric oxide) and potentiates vascular inflammatory response [31]. In Maier et al.’s analysis [32], the relation between low magnesium interleukin-1 (IL-1), vascular cell adhesion molecule-1 (VCAM) and plasminogen activator inhibitor (PAI)-1 concentration was noted, followed by several transcripts’ modulations. Though magnesium involvement in atherosclerosis pathology is widely postulated, the pioneering characteristics of our analysis are related to atherosclerosis location.

The topography divides the left main into a left-descending artery (LAD) and a circumflex artery (Cx). The latter arises at a high angle that may play a significant role in blood rheology and possible active different processes in LAD, including pro-atherosclerotic reactions. As the haemodynamic difference between both arteries, the relationship we wish to highlight may be secondary to that phenomenon already observed in circulation and linked to sodium [33, 34]. Secondary to the characteristic topography of the Cx, the possible inflammatory activation, including monocyte/macrophage cells, may occur that would explain the increased content of potassium in our analysis. The potassium was found as an inflammasome-induced trigger in previous reports [35].

We also indicated the relation between Ferrum concentration and the severity of lumen narrowing by atherosclerotic lesions in circumflex coronary. In previous analysis [36], the iron capacity on active redox activation that leads to the reactive oxygen species (ROS) and lipid peroxidation accumulation was noted. Yan et al. [37] revealed the increased risk of major adverse cardiovascular and cerebrovascular events related to excessive low and high iron concentrations. In the analysis of Ozdemir et al. [30], the association between iron concentration and high syntax score was presented. The cross-sectional study revealed higher Ferrum levels in patients diagnosed with coronary disease compared to healthy individuals. In Vinchi et al. [38], the atherosclerosis aggravation related to iron overload was presented, indicating its amelioration by dietary restriction.

Nickel exposure is postulated as associated with increased cardiovascular risk [39]. Our analysis revealed a positive correlation between nickel hair scalp accumulation and left main and circumflex artery disease risk. In their review, Rehman et al. [32] indicated that heavy metals, including nickel exposure sourced by contaminated water, may cause cardiovascular disorders and diabetes by reactive oxygen species upregulation. On the contrary, Li et al. [32] did not confirm the adequate support between nickel exposure and increased cardiovascular outcomes, as previous studies presented statistically significant findings for long-term exposure to nickel that could not establish temporality despite their cohort study design.

Copper is one of the trace elements essential for supporting normal physiologic processes through cuproptosis [40] and is claimed to be one of the mediating processes in cardiovascular disease pathogenesis and progression. The regulatory mechanisms induced by copper haemostasis and related pathways may help improve cardiovascular management [41], as its deficiency was found to be related to increased coronary disease [42].

As the inflammatory backgrounds of atherosclerosis are postulated, including calcified plaque formation, the role of calcium is well established [43]. The calcification of soft tissue denegation followed by necrosis processes is driven by inflammatory cells, foam cells, and osteoblasts about excessive cytokines, selectins, and myeloperoxidase release.

In atherogenesis, circulating monocytes differentiate into macrophages and form foam cells after penetrating into vascular endothelial cells [44]. Arsenic is postulated to increase monocyte adhesion to endothelium [45]. The arsenic concentration in our analysis was related to the left-descending artery and circumflex artery disease. Previous studies [46] revealed the increased formation of ROS/RNS, including peroxyl radicals (ROO·), the superoxide radical, singlet oxygen and hydroxyl radical (OH·) via the Fenton reaction, resulting in oxidant-induced DNA damage secondary to arsenic exposure. Arsenic as [47] seafood, water- and fat-soluble organoarsenic compounds represent the environmental and dietary elements reported accumulating in human organisms. Nigara et al.’s [48] meta-analysis found increased heart disease mortality related to arsenic urine output. In Kaur et al.’s [49] comprehensive review, the role of arsenic in cardiovascular pathology was demonstrated in the co-existence of other factors, such as obesity or smoking, which was explained by metal metabolism.

Strontium possesses anti-fracture efficacy but is also claimed to increase thromboembolic risk [50]. In our analysis, we measured strontium hair scalp content and found a correlation with atherosclerosis lesions. Animal studies indicated the vasodilatory effect of strontium by stimulating nitric oxide production [51], which may support the adequacy of our findings.

To our knowledge, our study is the first report aimed at atherosclerosis location and severity of lumen reduction. We determined the possible association between trace elements as enzymatic co-factors and toxic accumulations in coronary artery atherosclerosis locations.

## Study Limitation

The study was performed on patients presenting chronic coronary syndrome who were electively admitted for coronary angiography. The study points out the weak to moderate correlation between atherosclerosis location in coronary bed and trace element concentration. All patients were on optimal dyslipidaemic and diabetic pharmacological therapy. Hair trace element analysis contrary to serum concentration is not standardized, and the reference content is not presented but the main value of the study is not based on their concentration but correlation.

## Conclusions

The possible explanation of different epicardial artery involvement by atherosclerotic processes may lay in their topography and blood rheological characteristics that induce different inflammatory reactions co-factored by specific trace elements. The trace element concentration in the hair scalp may correlate with particular coronary atherosclerotic involvement, including the severity of lumen reduction. This may indicate the missing link between the pathophysiological processes of atherosclerosis development and its location in coronary arteries.

## Data Availability

The dataset analysed during the current study is available in the corresponding author’s repository. It will be shared after a reasonable request by e-mail contact within three years following the publication.
